# Monitoring renal oxygenation status by near-infrared spectroscopy during ureterorenoscopy in children

**DOI:** 10.55730/1300-0144.5544

**Published:** 2022-07-09

**Authors:** Sema ŞANAL BAŞ, Meryem ONAY, Çiğdem ARSLAN ALICI, Umut ALICI, Baran TOKAR

**Affiliations:** 1 Department of Anesthesiology and Reanimation, Faculty of Medicine, Eskişehir Osmangazi University, Eskişehir, Turkey; 2 Department of Anesthesiology and Reanimation, Faculty of Medicine, Eskişehir Osmangazi University, Eskilşehir, Turkey; 3 Department of Pediatric Surgery, Division of Pediatric Urology, Faculty of Medicine, Eskişehir Osmangazi University, Eskişehir, Turkey; 4 Department of Pediatric Surgery, Division of Pediatric Urology, Faculty of Medicine, Eskişehir Osmangazi University, Eskişehir, Turkey; 5 Department of Pediatric Surgery, Division of Pediatric Urology, Faculty of Medicine, Eskişehir Osmangazi University, Eskişehir, Turkey

**Keywords:** Near-infrared spectroscopy, renal oxygenation, endourology, children

## Abstract

**Background/aim:**

Near-infrared spectroscopy (NIRS) monitoring demonstrates renal blood flow, perfusion, and oxygenation changes. This study aimed to evaluate the effects of pediatric endourological interventions (PEI) on regional oxygen saturation value (rSO_2_) using renal NIRS monitoring.

**Materials and methods**: Patients having bilateral inguinal surgery (group I), cystoscopy (group II), and ureterorenoscopy (group III), 20 patients in each group, were included in the study. NIRS values before induction (T0) and at 5 min (T5), 10 min (T10), 15 min (T15), 20 min (T20), 25 min (T25), 30 min (T30) of the surgical procedure, and at the postextubation (Tend) were determined. The amount of irrigation fluid was recorded in groups II and III. The ureterorenoscopy group was also evaluated as two subgroups, as group III-R with patients having a “20%↓rSO_2_” and as group III-NoR, not having a “20%↓rSO_2_”.

**Results:**

The mean total volume of irrigation was higher in group III, but the difference was not significant between the subgroups III-R and III-NoR. Renal rSO_2_ decreased significantly in T25, T30, and T-end values in group III. “20%↓rSO_2_” was seen in 1 patient in group II and 7 patients in group III. In the subgroups III-R, all patients had an obstructive pathology and significant preoperative hydronephrosis with a mean renal pelvis AP diameter of 21.1 ± 16.4 mm.

**Conclusion:**

Although rSO_2_ significantly improves postoperatively, our data may suggest that congenital and acquired obstructive pathologies with hydronephrosis, prolonged operative time with continuous irrigation, and instrument movement in a narrow lumen may increase intrarenal pressure and the risk of renal hypoxia in endourological interventions. Preoperative evaluation of kidney functions and a meticulously well-planned intervention can prevent possible complications.

## 1. Introduction

As a result of advances in the technology of endoscopes and auxiliary instruments, indications of ureterorenoscopy have increased in children. Ureterorenoscopy is essential equipment in the diagnosis and treatment of several pathologies in pediatric urology, and the potential harms and complications of the procedure should be investigated.

Insertion and retrieval movement of ureterorenoscope in obstructive pathologies such as large urinary tract calculi; irrigation-induced increase in intrarenal pressure; ureteral hemorrhage and edema caused by access sheath; and sepsis induced by instrumentation and absorption of fluid and bacteria may lead to renal injury during ureterorenoscopy [1–3]. Operating at low renal pressures during pediatric endourological interventions (PEI) is always needed to lower risks of renal damage and absorption of fluid and bacteria. The physiological intrarenal pressure is in the range 0–20 cm H_2_O, whereas pressures of over 20 cm H_2_O indicate obstruction [4,5]. To achieve better exposure and vision during the procedure, increasing the flow and the pressure of irrigation might be needed, and that may cause an irrigation-induced increase in intrarenal pressure. Irrigation flow and pressure are not the only factors causing an increase in pressure. We may define other factors according to the Poiseuille equation. Ureterorenoscope occupying and narrowing the lumen with back and forth movement, increase in urine viscosity especially during lithotripsy, the length and the diameter of ureter also contribute to the pressure change during ureterorenoscopy [5,6]. Experimental studies showed that high pressures may cause renal injuries and complications such as forniceal rupture, retroperitoneal edema, vacuolization and degeneration of renal tubules, pericalyceal vasculitis, and degeneration [2,7]. Potential harms and complications of ureterorenoscopy in children have not been described well.

Regional oxygen saturation (rSO_2_) reflects the microcirculation of arterial, venous, and capillary vessels. Near-infrared spectroscopy (NIRS) is a noninvasive technique that monitors rSO_2_ reflecting perfusion status. NIRS measures rSO_2_ by interpreting oxyhemoglobin and deoxyhemoglobin signals that come back by transmitting near-infrared light to tissues. NIRS has the ability to continuously and simultaneously monitor tissue perfusion in different organ systems at the bedside without interrupting routine care. Researches have demonstrated its benefit in monitoring cerebral, intestinal, and renal perfusion to detect potential ischemic episodes [8–13]. NIRS measurement may reduce risks associated with many diseases that may lead to ischemic injury.

NIRS continuously and simultaneously monitors rSO_2_, by probes placed on different areas of the body such as the forehead (cerebral), abdomen (mesentery), and lower back (renal). Multiple factors may affect NIRS values, but the two main determinants are the tissue perfusion and tissue oxygen utilization [9–14]. Thus, as a noninvasive monitorization method, NIRS is important in demonstrating the detection of subclinical hypoperfusion, tissue saturation falls, and complications in several organs. Perioperative NIRS was used not only for cerebral monitorization during cardiac surgeries, it was also preferred to follow up renal blood flow and in the early detection of renal pathologies and serious clinical conditions, such as renal failure. There are studies in which renal NIRS contributes to the monitorization of variations in renal parameters and early detection of renal injuries associated with pathological rSO_2_. Comparing to baseline values, a 20% reduction in renal oxygenation was considered as renal hypoxia [10–16].

In children, the effect of endourological interventions on renal perfusion has not been reported yet. In this study, we aimed to evaluate the impact of ureterorenoscopy on renal oxygenation by NIRS.

## 2. Materials and methods

The study was performed prospective observational, randomized trial, with the approval of the University Clinic Research Ethics Committee (date/number: 10.12.2019/13). The patients were randomized into 3 groups with the use of a computer program number. ASA I-III pediatric patients between 1 month to 12 years of age, who had bilateral inguinal hernia repair or orchidopexy (group I), cystoscopy (group II), and ureterorenoscopy (group III) were included in the study. Ultrathin semirigid ureterorenoscope 4.5/6.5 Fr was used in ureterorenoscopy group. The patients having flexible ureterorenoscopy were not included into the study. Other exclusion criteria were the history of renal failure, renal tumors, infection of the perirenal region; receiving colloid or blood transfusion, having abscess, body mass index (BMI) not in the normal limits (≤3% or ≥97%) and abnormality in preoperative renal function tests [17]. Newborn patients were also excluded due to the possible effect of the patient’s size on the accuracy of the measurement. The number of patients included in the study was 60.

Heart rate (HR), noninvasive mean blood pressure (MBP), peripheral oxygen saturation (SpO_2_), and end-tidal carbon dioxide (etCO_2_) were monitored. Renal NIRS monitorization was also performed. Pediatric-sized oximetry sensors were placed on the left and right posterolateral side below the costovertebral angle overlying the kidney for renal NIRS monitorization. Changes in regional oxygen saturation values (rSO_2_) were monitored using by NIRS probe (INVOS 5100 Oximeter, Somanetics Corporation). NIRS provides clinicians an estimate of local tissue oxygen utilization by assessing post-capillary oxygenation. This system provides a broad spectrum of NIRS light wavelengths (685–870 nm) to estimate the ratio of mean tissue oxyhemoglobin or deoxyhemoglobin to provide accurate tissue oxygen saturation [11].

Patients’ ages, heights, body weights, body mass index (BMI), preoperative hemoglobin (Hb) and hematocrit (Htc), preoperative and postoperative blood urea nitrogen (BUN) and serum creatinine (sCr) levels, operation durations, drugs administered through the operation and bilateral renal rSO_2_ values were recorded.

The renal NIRS probes were placed below the costal margin and above the iliac crest with the emitting tip of the sensor lateral to the spine and the reading side of the sensor wrapping around the patient’s flank at the level of T10-L2. Each probe consists of a light source and 2 photodetectors to measure tissue oxygen levels at different tissue depths. After confirming the locations of renal NIRS probes, the average score was recorded as baseline value. Percentage changes in renal NIRS values and hemodynamic data were recorded simultaneously. Reductions of 20% or more in renal rSO_2_ were considered to be significant. Comparing to baseline values, a 20% reduction in renal oxygenation was considered as renal hypoxia [11]. Several studies have shown a reduction in renal oxygenation by rSO_2_ measured by NIRS [8,9,14]. Here, we considered renal NIRS values of reduction ≥ 20% is pathological.

All patients induction was performed with sevoflurane (50% oxygen and 50% nitrous protoxide), 2–3 mg/kg propofol and 1 mcg/kg remifentanil, and laryngeal mask airway was inserted. Fluid management was performed on all patients with the same perioperative protocol. Patients were adjusted to mechanical ventilation settings in the same mechanical ventilation device (Avence CS2 Datex Ohmeda, Madison WI, USA). Maintenance of anesthesia was provided with 50% oxygen/50% nitrous protoxide, 2%–4% sevoflurane (MAC 1–1.3) through 4 L/min fresh gas flow. Total fluid volume (sodium chloride 0.9%) used for irrigation throughout the procedure was recorded in groups II and III.

Right and left renal rSO_2_, HR, MBP, SpO_2_, and etCO_2_ values were recorded before induction (T0) and at 5 min (T5), 10 min (T10), 15 min (T15), 20 min (T20), 25 min (T25), 30 min (T30) of the surgical procedure, and at the postextubation (Tend). Perioperative hypoxia (SpO_2_ ≤ 90%), bradycardia (≤80 beats/min for infants, ≤70 beats/min for older children), hypotension (20% lower than the baseline blood pressure) were recorded. Atropine was delivered iv 0.01 mg/kg to patients with bradycardia, and ephedrine was administered 0.1 mg/kg to hypotensive patients if an increase in iv fluid replacement was not effective.

Group III (Ureterorenoscopy group) was also evaluated as two subgroups, as group III-R with patients having a significant reduction in renal rSO_2_ and as group III-NoR with patients not having that reduction. Those two subgroups were compared according to the patient’s age, sex, preoperative mean anteroposterior (AP) diameter of the renal pelvis on ultrasonography, and the total amount of irrigation fluid volume as ml/kg (sodium chloride 0.9%) administered through the ureterorenoscopy. To minimize the risk of injury to the ureteral mucosa as the ureterorenoscope was introduced into the ureter, the urinary bladder was verified to be empty.

### 2.1. Analysis of statistical data

SAS University Edition was used in the implementation of the analysis (SAS version 9.2 software, SAS Institute, Cary, NC, USA). Continuous data was given as mean ± standard deviation in the statistical analysis of data. Categorical data were given in percent (%). Shapiro-Wilk test was used to examine data conformance to normal distribution. Two-way repeated measurements ANOVA (one factor repeated) test was used for repeated measurements. For the determination of the direction and magnitude of the relationship between variables (correlation), Pearson correlation coefficients were calculated for normal distribution variables. Pearson’s chi-square and Pearson’s exact chi-square analyses were used in the analysis of crosstab, p < 0.05 value was accepted as a criterion for statistical significance. When posthoc power analysis was performed using the values received at the end of our study, the power of the study was found to be 0.8 in case there are 20 patients in each group and an alpha value of 0.05 was considered [18].

## 3. Results

The data of 60 children were evaluated. The mean age was 76.8 ± 16 months, mean weight was 20.5 ±2.7 kg, height was 107.8 ± 6 cm, and mean BMI was 15.67 ± 0.4 kg/m^2^. There was no statistically significant difference between age, weight, height, hemoglobin level, and BMI distributions of the groups (Table 1). The number of male patients was prominent in group I comparing to groups II and III.

Preoperative and postoperative blood urea nitrogen (BUN) and serum creatinine (sCr) levels were compared. Preoperative BUN and sCr levels were higher in group III comparing to the other groups (Table 1), but preoperative and postoperative values were within normal physiological limits.

The average duration of operation was 80.16 ± 30.02 min in group I; 32.13 ± 4.19 min in group II, and 61.21 ± 35.07 min in group III. This difference in operation times was statistically significant (p < 0.001).

Each group was also evaluated according to the change observed in renal rSO_2_ from the basal to T30 values. While a statistically significant difference was not found in the comparison of the basal and other time values of groups I and II; in group III, a significant progressive reduction in renal rSO_2_ from the basal value to T30 was determined (Table 2). In group III, the lowest renal rSO_2_ value was at T30.

**Figure 1:**
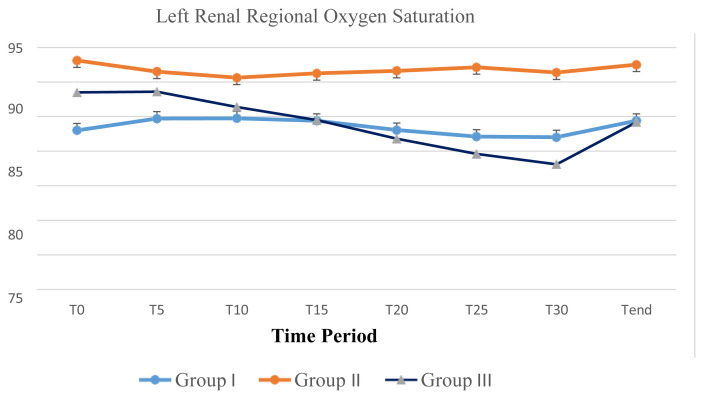
Changes in left renal regional oxygen saturation index during perioperative period.

**Figure 2:**
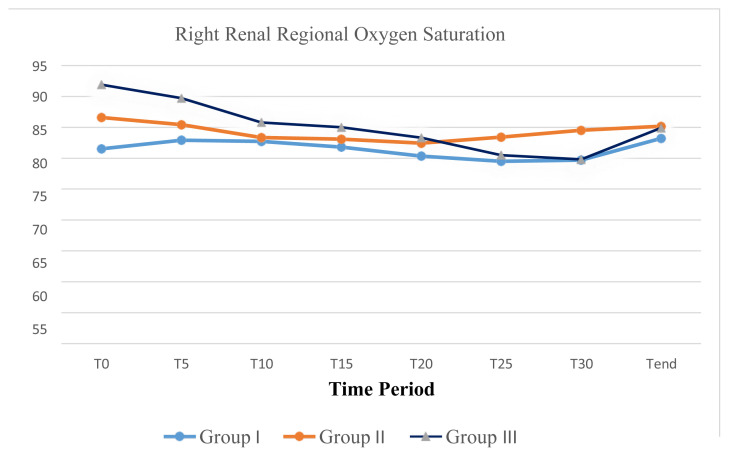
Changes in right renal regional oxygen saturation index during perioperative period.

In group III, when the decrease in renal rSO2 was evaluated according to the comparison of the sides where ureterorenoscopy was introduced; renal oxygenation measured by NIRS significantly decreased at the ipsilateral side where ureterorenoscopy was performed. The response to ureterorenoscopy by a reduction in renal rSO_2_ was more prominent on the right side. While a significant reduction comparing to the basal value was determined at T20, T25, and T30 of the left side ureterorenoscopy; the right side ureterorenoscopy caused a significant decrease in renal rSO_2_ at T10, T15, T20, T25, and T30 (Tables 2 and 3), (Figures 1 and 2). The reduction in renal rSO_2_ recovered at Tend in all patients of group III to the basal values except case 1 (Table 3), (Figures 1 and 2).

In our study, a significant reduction in renal rSO_2_ was observed in 8 patients. One in group II (5% of group II) and seven in group III (35% of group III). The range of decrease in renal rSO_2_ was between 20% and 26% (Table 3). The patient having a significant reduction in group II had cystoscopy, retrograde pyelography, and JJ stent insertion at the right side. She had a previous right pyeloplasty operation and the renal pelvis AP diameter was 25 mm before the cystoscopy. In group III, a significant reduction in renal rSO_2_ was observed at the ureterorenoscopy side in 7 patients. Only one patient had a bilateral decrease during the procedure (case I in Table 3), and the same patient was the only one in group III whose Tend value did not recover well, staying significantly lower than the basal value. The other 6 patients had an increase in renal rSO_2_ at Tend.

In group III, 17 patients out of 20 had ureterorenoscopy for Ho:YAG laser lithotripsy (the laser fiber size was 365 μm). The other 3 patients had ureterorenoscopy for ureteropelvic polyp, ureteral stenosis, and postpyeloplasty ureteropelvic junction obstruction. The patient having ureteropelvic polyp had a diagnostic ureterorenoscopy with a preoperative diagnosis of renal stone. Ureterorenoscopy revealed that on the ureteropelvic junction he has a polyp with protruding arms into the pelvis and ureter. Laparoscopic excision of the polyp and pyeloplasty were performed. All patients in group III had unilateral ureterorenoscopy. Double J stent was placed postoperatively in 8 patients out of 17 ureteroscopic lithotripsy and 2 patients who had ureterorenoscopy and dilatation.

The data of group III were evaluated also with the comparison of two subgroups. While subgroup III-R (n = 7) had patients with a 20% or more reduction in renal rSO_2_ during ureterorenoscopy, patients in subgroup III-NoR (n = 13) had renal rSO_2_ values in acceptable limits (Table 4). Of 7 patients in subgroup III-R, 6 patients had ureterorenoscopy for lithotripsy and one for ureteral stenosis. The patient having a bilateral decrease in renal rSO_2_ and not recovering well at Tend had lithotripsy for an obstructing proximal ureteral stone. The renal rSO_2_ value returned to basal value in postoperative follow-up of this patient. There was a significant difference for the mean age and preoperative renal pelvis AP diameters in comparison of the subgroups. The patients in subgroup III-R were older in comparison to subgroup III-NoR. Hemodynamic data, renal pathology and NIRS values of the patients were statistically compared according to age correlation. All patients were older than 8 years old except 2 patients and had significant hydronephrosis with a mean renal pelvis AP diameter of 21.1 ± 16.4 mm in subgroup III-R (Table 4).

**Figure 3:**
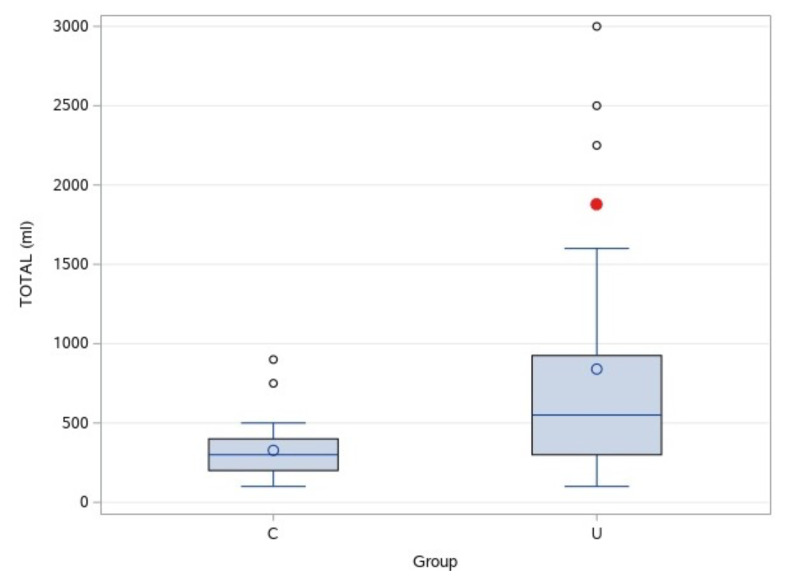
Irrigation fluid of group II (C) and group III (U).

Nine-tenth percent saline irrigation was used during cystoscopy in group II and ureteroscopy in group III. While the mean total volume of irrigation in group II was 329.16± 188.4 mL (20.10 ± 8.16 mL/kg), it was 840.0 ± 185.5 mL (41.75 ± 8.03 mL/kg) in group III. The difference for the mean values between groups II and III was statistically significant (p: 0.0114) (Figure 3). In the intragroup analysis of group III, the difference for the mean total volume of irrigation was not significant between the subgroups III-R and III-NoR (Table 4).

Hemodynamic parameters were stable during the surgical interventions and there was no statistically significant difference in the comparison of the three groups in terms of HR, MBP, SpO_2_, and etCO_2_ (HR p: 0.772, MBP p: 0.339, SpO_2_ p: 0.6636, etCO_2_ p: 0.688). In the intragroup analysis, HR, MBP, SpO_2_, and etCO_2_ from T5 to Tend were compared with T0 (the basal value) in each group and the difference found was not significant (Table 4).

## 4. Discussion

The use of renal tissue oxygenation monitoring during anesthesia and surgery has been extensively described in the pediatric patient. While rSO2 shows the balance between oxygen requirements in tissues and actual tissue oxygenation, it also reflects the microcirculation of arterial, venous, and capillary vessels. Factors such as anemia, hypotension, bradycardia, hypercapnia, and hypoxia affect the rSO_2_ value so that preoperative hemoglobin was recorded and perioperative hemodynamic parameters were followed. There was no statistically significant difference between age, weight, height, hemoglobin level, and BMI distributions of the groups, but the patients in subgroup III-R were significantly older and had a significant hydronephrosis with a mean renal pelvis AP diameter of more than 20 mm. There was no 20% or more reduction in renal oxygenation of patients operated for inguinal hernia and orchidopexy in group I. The only patient who had a significant reduction during cystoscopy in group II, had a UPJ obstruction due to a previous pyeloplasty operation and a significant hydronephrosis. While subgroup III-R had patients with a 20% or more reduction in renal rSO_2_ during ureterorenoscopy, patients in subgroup III-NoR had renal rSO_2_ values in acceptable limits. Of 7 patients in subgroup III-R, 6 patients had ureterorenoscopy for lithotripsy and one for ureteral stenosis. Another finding in our study was that the average duration of operation was significantly longer in group III comparing to group II. Renal oxygenation measured by NIRS significantly decreased at the ipsilateral side where ureterorenoscopy was performed. The reduction was significantly more prominent at T25 and T30 for both left and right sides in group III. This may indicate that ureterorenoscopy with a longer duration, especially after the first 25 min may disturb perioperative renal oxygenation in children.

NIRS may detect subclinical hypoperfusion and dysfunction of microcirculation with tissue saturation falls in several organs [9–18]. Renal NIRS monitoring should be considered to be included in prognostic parameters, especially in patients who have a risk for renal injury. Monitoring of renal rSO_2_, guiding interventions to prevent or treat a low renal rSO_2_, will improve renal outcome after surgery in the selected patients [19].

As a noninvasive procedure, renal NIRS monitorization may provide early detection of renal injuries associated with pathological rSO_2_ and it could be applied in intensive care follow-up or in invasive procedures that may affect the perfusion of the kidney [8–13]. Anemia, hypotension, and change in the partial pressure of carbon dioxide may affect the reliability of renal NIRS monitorization [14]. In our study, HR, noninvasive MBP, SpO_2_, etCO_2_ Hb, and Htc were no significant change that would affect renal NIRS monitorization was observed. Renal rSO_2_ decreased significantly in T25, T30, and T-end values in group III. “20%↓rSO_2_” was seen in 1 patient in group II and 7 patients in group III. A significant postoperative increase in T-end value of renal rSO_2_ was observed in all patients except one. In the subgroups III-R, all patients had an obstructive pathology and significant preoperative hydronephrosis with a mean renal pelvis AP diameter of 21.1 ± 16.4 mm.

The kidney is one of the organs highly sensitive to hypoxic changes. NIRS monitoring has been used to detect oxygen saturation changes in renal tissue [11–15]. Continuous and instantaneous measurements of renal tissue perfusion can be performed noninvasively with NIRS monitoring. Several studies have shown a reduction in renal oxygenation by rSO_2_ measured by NIRS [10,11,16]. Basal values and reductions differ in renal oxygenation studies with NIRS. In a study, it was stated that 65% is the limit value of renal oxygenation for rSO2, and values lower than this and also reductions of 25% could be considered to be an alert for renal hypoxia [10]. A 20% reduction of rSO_2_ from the baseline values was considered to be significant in studies examining acute renal damage during cardiopulmonary bypass [11]. In another study, values of 20% were considered to be clinically significant and 50% rSO_2_ value was considered to be pathological for all measurements [16]. A change of 10% during the evaluation of renal oxygenation was also accepted as clinically significant saturation changes [18]. We considered reduction ≥ 20% in renal NIRS values pathological in our study.

Comparing to baseline values, a 20% reduction in renal oxygenation was considered as renal hypoxia [11,16]. Critical decreases in renal saturation values might be associated with acute kidney injury [9–11]. In our study, there was no 20% or more reduction in renal oxygenation of patients operated for inguinal hernia and orchidopexy in group I. The only patient who had a significant reduction during cystoscopy in group II, had a UPJ obstruction due to a previous pyeloplasty operation and a significant hydronephrosis. A reduction in renal oxygenation occurred at the same side where retrograde pyelography and JJ stent insertion were done and it was prominent between 15 to 25 minutes of the procedure. In ureterorenoscopy group, all 7 patients with a significant decrease in renal rSO_2_ had common findings that were significant hydronephrosis with a mean value of 21.1 ± 16.4 mm and intervention at the ipsilateral side where rSO_2_ drops. Our findings in groups II and III may suggest that endoscopic intervention in obstructive pathologies with hydronephrosis might cause ipsilateral renal hypoxia during the procedure. The renal rSO_2_ returned to basal value at the end of the procedure in all patients except one with the bilateral and persistent drop at Tend. This patient had obstructive proximal ureteral stone and recovered in postoperative follow-up. Although renal rSO_2_ in our patients recovered well postoperatively, our data may also suggest that if endoscopic intervention is required in patients having obstructive pathologies with hydronephrosis, preoperative evaluation of kidney functions and a meticulously well-planned intervention can prevent possible kidney damage.

Achieving good vision and ergonomics in endourologic intraluminal procedures in the ureter and renal pelvis is a challenging process in children. Potential renal damages caused by endourological interventions in children have not been investigated well in the literature. Smaller intraluminal diameter in children associated with congenital urinary system abnormalities and acquired obstructive pathologies may contribute to intrarenal pressure change and complications during the interventions [5,6,20]. Renal complications due to a high intrarenal pressure were shown by experimental studies [2,7]. Factors narrowing the lumen and increasing the intrarenal pressure over 20 H_2_O during endourological procedures in children may cause renal damage [4,5]. Perioperative renal hypoxia shown in our study might be related to an increase in renal pressure. Congenital and acquired obstructive pathologies such as ureteropelvic junction obstruction, ureteral stenosis or urinary tract calculi; back and forth movement of instruments, volume and flow rate of irrigation fluid through the procedure and increase in urine viscosity especially during lithotripsy may increase intrarenal pressure and contribute to renal hypoxia in endourological interventions. In our study, all patients with a significant reduction in renal rSO_2_ had a congenital or acquired obstructive pathology narrowing the lumen and associated with hydronephrosis.

The first group with inguinal operations had the longest average surgical duration in our study. In the comparison of the surgical duration of groups II and III, the duration was significantly longer in ureterorenoscopy group (group III). These findings may suggest that duration of operation did not have a direct effect on renal rSO_2_, but a longer duration in endourological interventions may alter intrarenal pressure and renal oxygenation due to longer exposure to continuous irrigation and back and forth movement of instruments in a narrow space. In our study, 75% of the patients with a significant reduction in renal rSO_2_ had urinary tract calculi. This may also suggest a higher urine viscosity due to lithotripsy may also contribute to intrarenal pressure change.

Renal oxygenation measured by NIRS significantly decreased at the ipsilateral side where ureterorenoscopy was performed. The reduction was significantly more prominent at T25 and T30 for both left and right sides in group III. This may indicate that ureterorenoscopy with a longer duration, especially after the first 25 min may disturb perioperative renal oxygenation in children. Our data may suggest that longer the duration of ureterorenoscopy or any endoscopic intraluminal procedure in children increases the risk of renal hypoxia and procedures longer than 25 min with excessive instrument movement should be avoided. The same measures might also be considered in percutaneous nephrolithotomy.

The response to ureterorenoscopy by a reduction in renal rSO_2_ was more prominent on the right side. While a significant reduction comparing to the basal value was determined at T20 to T30 of the left side ureterorenoscopy; the right side ureterorenoscopy caused a significant decrease in renal rSO_2_ at T10 to T30. There was no significant anatomical or pathological difference between the right and the left side that would explain the earlier reduction in renal rSO_2_ on the right side.

The sample size limited with 60 patients and lack of previous research studies on the topic were the limitations of the study. Although we have these limitations our study may start a discussion on important questions, “How endourological interventions in children affect renal rSO_2_ and whether renal NIRS may contribute to the monitorization of renal rSO_2_ and early detection of renal injuries”. The well-defined structure of our study would provide us and the other physicians a base to perform prospective studies with an increased number of patients. Those studies should be planned to define an algorithm that could serve to determine the patients who need renal NIRS monitorization and to establish a protocol to prevent renal injury during PEI. In order to minimize the threat of inclusion biases in the further researches, the size of the data set and duration of the study need to be increased. There was no statistically significant difference between age, weight, height, hemoglobin level, and BMI distributions of the groups, but the patients in subgroup III-R were significantly older and had a significant hydronephrosis with a mean renal pelvis AP diameter of more than 20 mm. Another finding in our study was that the average duration of operation was significantly longer in group III comparing to group II. In the further researches, higher number of patients should be evaluated by grouping them according to different age groups, short or long duration of surgery, and the grade of hydronephrosis. Another topic that might be considered as one of the future prospective studies is the sonographic Doppler renal resistive index evaluation correlated with the NIRS for renal rSO_2_.

In conclusion, PEI especially ureterorenoscopy may adversely affect renal oxygenation in children. However, renal NIRS values returned to the nearly normal range at the end of the procedure. Our result may suggest that PEI might reduce renal oxygenation without causing any pathophysiological changes in heart rate and blood pressures. The kidney is highly sensitive to hypoxic changes. The data of the study may also suggest that if PEI is required in patients having congenital or acquired obstructive pathologies and significant hydronephrosis, a drop in renal rSO_2_ may occur depending on the duration of the procedure and the factors that may increase the intrarenal pressure such as excessive back and forth movement of instruments, high volume and flow rate of irrigation fluid and increase in urine viscosity especially during lithotripsy. Renal injury has a complex pathophysiology with a number of different predictors and pathophysiologic mechanisms and also acute kidney injury may occur in the perioperative period so that NIRS monitoring, which can detect changes in renal rSO_2_, has been shown to be safe and feasible. Therefore, renal NIRS may detect early renal rSO_2_ changes and predict the development of kidney ınjury. Thus, renal NIRS monitoring should be considered to be included in prognostic parameters, especially in patients who have a risk for renal injury. It may improve renal outcome after surgery and decrease perioperative and postoperative morbidity and mortality in the selected patients.

## Figures and Tables

**Table 1:** Demographic and clinical data in groups.

Parameters	Group I (n = 20)	Group II (n = 20)	Group III (n = 20)	p levels
Age (month)	78.3 ± 12.6	79.3 ± 21.7	72.8 ± 13.8	0.640
Sex (Female/male)	3/17	8/12	11/9	0.023*
Weight (kg)	20.6 ± 2.5	21.2 ± 2.8	19.9 ± 2.8	0.387
Height (cm)	105.05 ± 5.8	110.21 ± 8.2	110 ± 8.2	0.457
Body mass index (kg/m^2^)	15.11 ± 0.3	16.2 ± 0.5	15.7 ± 0.4	0.308
BUN (mg/dL)	0.37 ± 0.26	0.18 ± 0.4	1.37 ± 0.3*	0.0002*
sCr (mg/dL)	0.01 ± 0.029	0.03 ± 0.04	0.18 ± 0.41*	0.0005*
Hemoglobine (g/dL)	12.5 ± 0.2	12.3 ± 0.3	12.2 ± 0.37	0.833
Hematocrit (%)	33.7 ± 1.2	36.9 ± 1.8	37.1 ± 1.8	0.932

BUN: blood urea nitrogen, sCr: serum creatinine levels.

Data are presented as number of patients (%) or mean ± standard error.

* p < 0.05 indicates statistically significant values.

**Table 2:** Comparison of renal regional oxygen saturation index in groups.

	*Group I*		*Group II*		*Group III*		*Group III (p* ^#^ *)*		*Comparison of intergroup (p* ^*^ *)*	
*Time*	*Left rSO* *_2_* * (%)*	*Right rSO* *_2_* * (%)*	*Left rSO* *_2_* * (%)*	*Right rSO* *_2_* * (%)*	*Left rSO* *_2_* * (%)*	*Right rSO* *_2_* * (%)*	*Left rSO* *_2_*	*Right rSO* *_2_*	*Left rSO* *_2_*	*Right rSO* *_2_*
*T0-5*	2 ± 1.0	2.1 ± 0.9	−1.8 ± 1.6	−1.0 ± 1.3	−2.6 ± 1.4	−3.0 ± 1.4^#^	p = 0.93	p = 0.0156^#^	p = 0.41	p = 0.11
*T0-10*	2.3 ± 1.3	2.1 ± 1.4	−2.5 ± 2.1	−2.1 ± 1.77	−2.5 ± 1.9	−4.9 ± 1.9^#^	p = 0.021	p = 0.028^#^	p = 0.27	p = 0.46
*T0-15*	1.7 ± 1.5	1.8 ± 1.3	−2.0 ± 2.4	−1.9 ± 2.4	−4.2 ± 2.1	−5.5 ± 2.1	p = 0.54	p = 0.034^#^	p = 0.51	p = 0.65
*T0-20*	0.2 ± 1.6	0.4 ± 2.1	−1.8 ± 2.6	−2.6 ± 2.1	−7.8 ± 2.4^#^	−5.9 ± 2.4^#^	p = 0.0025^#^	p = 0.028^#^	p = 0.054	p = 0.99
*T0-25*	−0.8 ± 1.7	−0.7 ± 2.1	−1.8 ± 2.7	−1.8 ± 2.2	−10 ± 2.4^#*^	−8.7 ± 2.4^#*^	p = 0.0003^#^	p = 0.0004^#^	p = 0.027^*^	p = 0.025^*^
*T0-30*	−0.8 ± 1.6	−0.8 ± 1.5	−1.7 ± 2.6	−0.16 ± 2.1	−11.2 ± 2.3^#*^	−9.4 ± 2.3^#*^	p<0.0001^#^	p = 0.0026^#^	p = 0.02^*^	p = 0.009^*^
*Tend*	1.8 ± 3.1	1.9 ± 2.7	0.16 ± 4.9	0.1 ± 4.0	−5.7 ± 4.3^#*^	−6.8 ± 4.3^#*^	p = 0.033^#^	p = 0.0014^#^	p = 0.02^*^	p = 0.045^*^

* p: comparison between of intergroup,

* p < 0.05 indicates statistically significant.

# p: comparison of intragroups,

# p < 0.05 indicates statistically significant values.

rSO2: regional oxygen saturation index.

**Table 3:** The patients with a significant reduction in renal rSO2.

	Case 1		Case 2		Case 3		Case 4		Case 5		Case 6		Case 7		Case 8	
	*Left rSrO* *_2_* * (%)*	*Right rSrO* *_2_* * (%)*	*Left rSrO* *_2_* * (%)*	*Right rSrO* *_2_* * (%)*	*Left rSrO* *_2_* * (%)*	*Right rSrO* *_2_* * (%)*	*Left rSrO* *_2_* * (%)*	*Right rSrO* *_2_* * (%)*	*Left rSrO* *_2_* * (%)*	*Right rSrO* *_2_* * (%)*	*Left rSrO* *_2_* * (%)*	*Right rSrO* *_2_* * (%)*	*Left rSrO* *_2_* * (%)*	*Right rSrO* *_2_* * (%)*	*Left rSrO* *_2_* * (%)*	*Right rSrO* *_2_* * (%)*
T0	92 (0)	88 (0)	84 (0)	75 (0)	94 (0)	95 (0)	95 (0)	95 (0)	90 (0)	88 (0)	91 (0)	89 (0)	93 (0)	95 (0)	79 (0)	74 (0)
T5	91 (−1)	88 (0)	86 (2)	75 (0)	93 (−1)	95 (0)	85 (−11)	95 (0)	83 (−7)	82 (−5)	86 (−6)	85 (−4)	83 (−8)	89 (−6)	74 (−5)	66 (−11)
T10	90 (−2)	86 (−2)	81 (−4)	74 (−1)	92 (−2)	95 (0)	84 (−12)	90 (−5)	76 (−16)	78 (−14)	82 (−10)	82 (−5)	75 (−14)	86 (−10)	73 (−8)	63 (−15)
T15	90 (−2)	84 (−3)	82 (−2)	74 (−1)	87 (−7)	95 (0)	83 (−13)	91 (−4)	73 (−18)	76 (−16)	77 (−14)	86 (−3)	75 (−14)	84 (−12)	70 (−11)	61 (−18)
T20	89 (−3)	79 (−10)	73 (−13)	69 (−8)	76 (−20)	95 (0)	79 (−17)	85 (−11)	73 (−18)	71 (−22)	75 (−18)	85 (−4)	76 (−12)	80 (−14)	69 (−13)	59 (−20)
T25	63 (−23)	68 (−24)	67 (−20)	69 (−8)	72 (−22)	94 (−1)	75 (−22)	88 (−10)	75 (−15)	69 (−23)	72 (−20)	87 (−2)	80 (−10)	76 (−20)	69 (−13)	61 (−18)
T30	58 (−25)	62 (−25)	68 (−20)	67 (−9)	76 (−20)	95 (0)	76 (−20)	90 (−5)	80 (−10)	72 (−20)	71 (−22)	88 (−1)	88 (−4)	77 (−18)	77 (−2)	67 (−10)
Tend	61 (−24)	65 (−26)	81 (−4)	80 (7)	82 (−11)	95 (0)	85 (−11)	91 (−4)	84 (−6)	80 (−7)	83 (−10)	89 (0)	90 (−3)	84 (−12)	77 (−2)	67 (−10)

rSO2: regional oxygen saturation.

Cases 1–7: in group III, case 8: in group II.

**Table 4:** Age, sex, preoperative renal pelvis AP diameter and total amount of irrigation fluid during ureterorenoscopy in group III.

Group III Subgroups	Mean age year ± SD (range)	Sex Female/male	Preoperative USG Mean AP diameter ± SD (mm) (range)	Total amount of irrigation fluid during URS (mL/kg) (range)
**Group III-R (n = 7)**	7.21 ± 4.26 (1–11)	4/3	21.1 ± 16.4 (9–57)	37.86 ± 29.7 (10–100)
**Group III-NoR (n = 13)**	4.4 ± 2.95 (1–10)	8/5	6.85 ± 8.71 (0–30)	39.6 ± 34.24 (10–125)

Group III-R: patients having ureterorenoscopy and having a 20% reduction in renal rSO_2_.

Group III-NoR: patients having ureterorenoscopy and not having a 20% reduction in renal rSO_2_.

**Table 5:** Comparison of the hemodynamic parameters in groups.

Heart rate (per beat/min)	Group I	Group II	Group III	p levels
T0	102.4 ± 3.9	104.9 ± 5.1	104.0 ± 5.6	0.22
T5	105.6 ± 3.5	106.6 ± 5.6	106.9 ± 5.0	0.72
T10	106.1 ± 3.6	107.2 ± 4.6	109.7 ± 5.1	0.56
T15	108.8 ± 3.8	108.3 ± 6.1	109.9 ± 5.4	0.62
T20	107.4 ± 3.4	106.0 ± 4.4	107.4 ± 4.7	0.65
T25	107.6 ± 3.3	105.0 ± 4.3	106.4 ± 4.7	0.87
T30	107.1 ± 3.5	104.5 ± 4.5	104.4 ± 4.9	0.53
Tend	108.3 ± 3.6	107.5 ± 4.6	106.2 ± 5.0	0.48
Mean blood pressure (mmHg)	**Group I**	**Group II**	**Group III**	**p levels**
T0	60.5 ± 3.0	61.7 ± 4.3	60.2 ± 4.2	0.60
T5	61.2 ± 2.8	62.8 ± 4.1	63.1 ± 4.1	0.88
T10	60.8 ± 2.4	61.5 ± 3.4	62.0 ± 3.3	0.78
T15	62.0 ± 2.5	62.0 ± 3.6	61.5 ± 3.9	0.51
T20	62.5 ± 2.3	63.1 ± 3.6	61.5 ± 3.6	0.62
T25	62.4 ± 2.6	62.8 ± 3.3	62.0 ± 3.3	0.92
T30	62.8 ± 2.7	63.9 ± 3.7	62.1 ± 3.7	0.59
Tend	66.4 ± 2.4	67.6 ± 3.4	66.4 ± 3.4	0.57
End-tidal CO_2_ (mmHg)	**Group I**	**Group II**	**Group III**	**p levels**
T0	31.7 ± 0.5	30.2 ± 0.7	30.4 ± 0.7	0.84
T5	32.5 ± 0.6	30.7 ± 0.8	30.8 ± 0.8	0.64
T10	33.5 ± 0.6	29.7 ± 0.9	30.6 ± 0.9	0.96
T15	32.7 ± 0.7	29.3 ± 1.0	30.2 ± 1.0	0.73
T20	33.1 ± 0.7	29.5 ± 1.0	30.1 ± 1.0	0.77
T25	32.7 ± 0.7	29.5 ± 1.0	29.8 ± 1.0	0.99
T30	32.0 ± 0.6	29.7 ± 0.9	29.5 ± 0.9	0.80
Tend	31.7 ± 0.6	29.9 ± 0.9	29.9 ± 0.9	0.74
Peripheral oxygen saturation (%)	**Group I**	**Group II**	**Group III**	**p levels**
T0	99.20 ± 0.19	99.12 ± 0.30	99.00 ± 0.27	0.83
T5	99.75 ± 0.11	99.50 ± 0.18	99.50 ± 0.16	0.22
T10	99.85 ± 0.09	99.87 ± 0.15	99.70 ± 0.14	0.89
T15	99.85 ± 0.11	99.75 ± 0.17	99.60 ± 0.16	0.20
T20	99.85 ± 0.12	99.87 ± 0.19	99.60 ± 0.17	0.23
T25	99.80 ± 0.11	99.87 ± 0.18	99.60 ± 0.16	0.31
T30	99.90 ± 0.10	99.87 ± 0.16	99.54 ± 0.14	0.10
Tend	99.80 ± 0.11	99.75 ± 0.18	99.61 ± 0.16	0.32

* p< 0.05 indicates statistically significant values.
